# Peripheral Nerve Regeneration and Muscle Reinnervation

**DOI:** 10.3390/ijms21228652

**Published:** 2020-11-17

**Authors:** Tessa Gordon

**Affiliations:** Department of Surgery, University of Toronto, Division of Plastic Reconstructive Surgery, 06.9706 Peter Gilgan Centre for Research and Learning, The Hospital for Sick Children, Toronto, ON M5G 1X8, Canada; tessat.gordon@gmail.com; Tel.: +1-(416)-813-7654 (ext. 328443) or +1-647-678-1314; Fax: +1-(416)-813-6637

**Keywords:** peripheral nerve injuries, peripheral nerve regeneration, skeletal muscle reinnervation, Schwann cells, regenerating peripheral nerves

## Abstract

Injured peripheral nerves but not central nerves have the capacity to regenerate and reinnervate their target organs. After the two most severe peripheral nerve injuries of six types, crush and transection injuries, nerve fibers distal to the injury site undergo Wallerian degeneration. The denervated Schwann cells (SCs) proliferate, elongate and line the endoneurial tubes to guide and support regenerating axons. The axons emerge from the stump of the viable nerve attached to the neuronal soma. The SCs downregulate myelin-associated genes and concurrently, upregulate growth-associated genes that include neurotrophic factors as do the injured neurons. However, the gene expression is transient and progressively fails to support axon regeneration within the SC-containing endoneurial tubes. Moreover, despite some preference of regenerating motor and sensory axons to “find” their appropriate pathways, the axons fail to enter their original endoneurial tubes and to reinnervate original target organs, obstacles to functional recovery that confront nerve surgeons. Several surgical manipulations in clinical use, including nerve and tendon transfers, the potential for brief low-frequency electrical stimulation proximal to nerve repair, and local FK506 application to accelerate axon outgrowth, are encouraging as is the continuing research to elucidate the molecular basis of nerve regeneration.

## 1. Introduction

Injured nerves in the peripheral nervous system, including motor and sensory nerves supplying muscles and sense organs respectively, have the capacity to regenerate and reinnervate their target organs, unlike the nerves in the central nervous system that do not [1,2,3,4,5,6].

### 1.1. Wallerian Degeneration

After the two most severe peripheral nerve injuries of six types, crush (axonotmesis) and transection (neurotmesis) injuries [6], the nerve fibers distal to the site of the injury lose their contact with the neuronal cell body. They are deprived, for all intents and purposes, of their source of synthesis of proteins, glycoproteins, lipids and carbohydrates [1,2,3,4,5,6]. As a result, the nerve fibers undergo Wallerian degeneration, leaving the connective tissue sheaths and the Schwann cell (SC)-containing basal lamina tubes intact [7]. The continued axonal transport sustains the distal stump in a proximo-distal direction for up to two days, allowing a continued capacity to conduct action potentials [8,9]. Live calcium imaging revealed an initial transient calcium influx that is localized primarily to the axotomy site. A slower rise in calcium influx progresses as a wave throughout the cytoplasm and the mitochondria of the distal stumps [10]. The calcium triggers endogenous proteolysis and the degeneration of the axonal cytoskeleton. This is followed by fragmentation, disintegration, and finally, the phagocytosis of the axons and their myelin, initially by the SCs themselves [11,12,13,14]. Thereafter, the macrophages that enter through the leaky blood–brain barrier, play the predominant role in the Wallerian degeneration [15,16,17,18], the duration of which is surprisingly long, up to a month in rats for example [17,19].

### 1.2. Denervated SCs are Growth-Supportive

**Figure 1:**
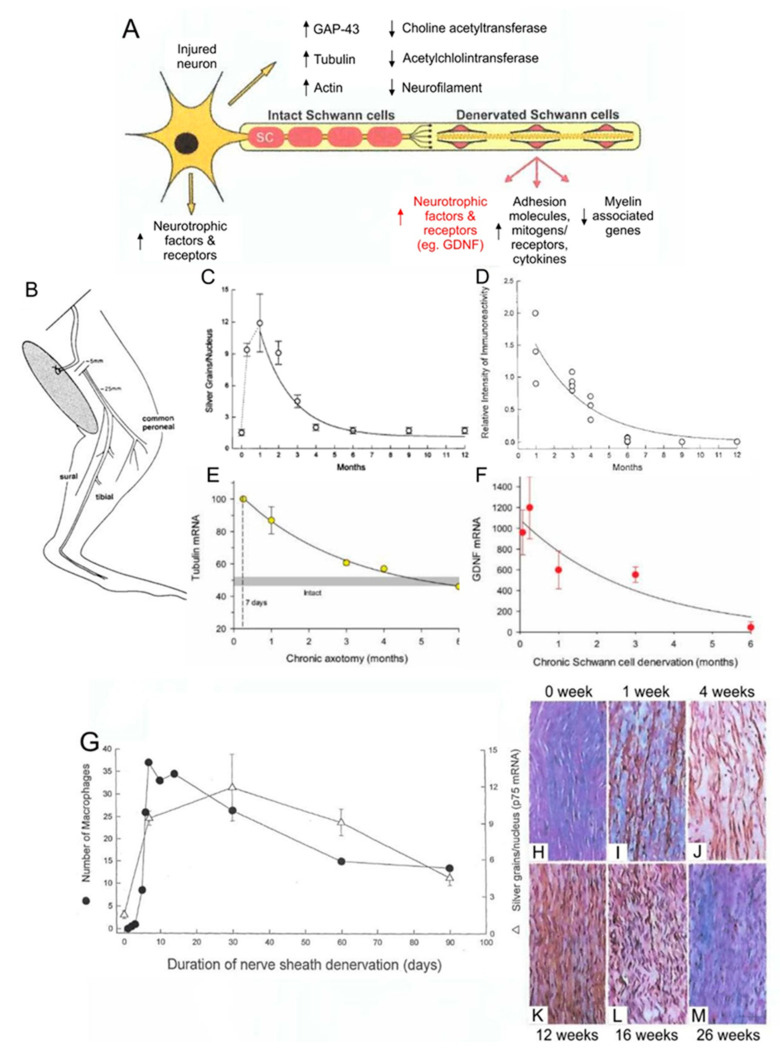
Expression of growth-associated genes in axotomized motoneurons after loss of target connections and in chronically denervated Schwann cells (SCs) in the distal nerve stumps. An illustrated motoneuron showing expression of growth-associated genes in motoneurons and in Schwann cells in the distal nerve stumps after a nerve injury (**A**). Experiments were performed in which we cut the sciatic nerve and sutured both proximal and distal nerve stumps to innervated muscle to prevent regeneration (**B**). p75NTFR in situ hybridization showing an early rise in expression in the denervated SCs in the distal nerve stump that declined within 6 months (**C**). Immunocytochemical evidence of a concomitant decline in p75 protein in the chronically denervated Schwann cells. (**D**). In situ hybridization showing a progressive decline to baseline values in the upregulation of the mRNA of the cytoskeletal protein tubulin in axotomized motoneurons (**E**). Semi-quantitation of the gene expression of Glial derived neurotrophic factor (GDNF) in the denervated distal nerve stump with rt-PCR, showing an exponential decline (**F**). The parallel expression of p75 and infiltration of macrophages into the denervated distal nerve stump (**G**). The slow rate of Wallerian degeneration of the isolated nerve in the denervated distal nerve stump is illustrated in the longitudinal micrographs of the denervated distal nerve stump (**H**–**J**) leaving parallel endothelial tubes with immunologically stained p75 expressing SCs by 12 weeks (**K**) that declines within 6-months (**L**,**M**). Adapted from [19].

**Figure 2:**
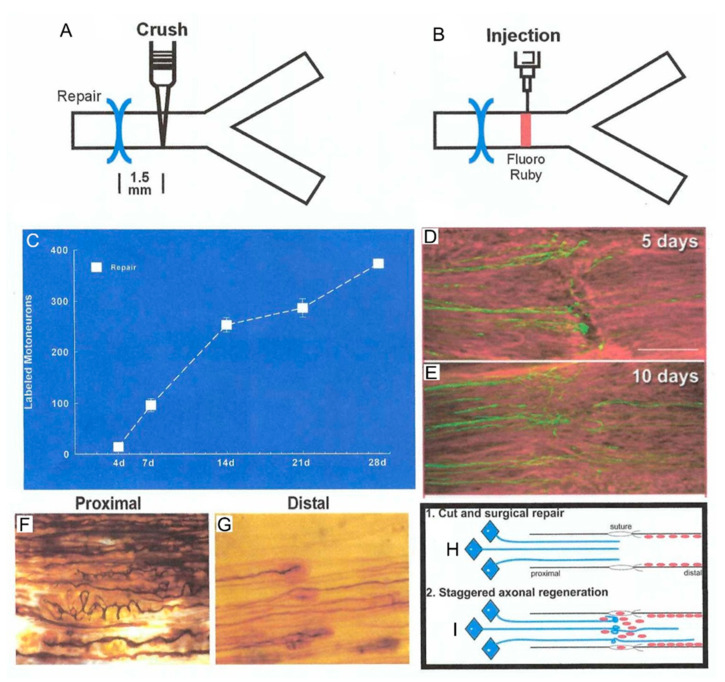
**Outgrowth of axons after femoral nerve transection and microsurgical repair.** The femoral neve was crushed 1.5 mm distal to the repair site (**A**) for rubyred microinjection (**B**) to count motoneurons that had regenerated their axons across the suture site. (**C**). The motoneuron count elucidated a staggering of the regenerating axons across the suture site with all axons crossing the site by 28 days, considerably longer than the calculated latent period of 2–3 days. The slow and staggered crossing the suture is seen in longitudinal sections of the nerve with axons of motoneurons immunostained for neurofilament protein, 5 (**D**) and 10 (**E**) days after the nerve repair. The complex growth of silver nitrate-stained regenerating axons (**F**) even turns back into the proximal nerve stump as first seen by Cajal [41]. The staggering of the regenerating axons within the endoneurial tube of the distal nerve stump is seen in (**G**). The cut and surgical repair is illustrated in (**H**,**I**). where regenerating axons “stagger” across the suture site in amongst the Schwann cells that move into the surgical site from both nerve stumps. The Schwann cells elongate along the basal lamina of the denervated endoneurial tubes to guide, support, and myelinate the regenerating axon. Adapted from [33].

As the denervated SCs dedifferentiate, proliferate, elongate, and line the endoneurial tubes within the fascicles of the denervated distal nerve stumps, they downregulate myelin-associated genes and upregulate growth-associated genes (Figure 1A,I–K) [17,20,21,22]. The former genes include *P*_0_, and the latter, the transcription factor, *c-Jun*, and the p75 neurotrophic factor receptor (*p75NTR*). Expression of c-Jun is critical for the induction of the repair function of the SCs [23], c-Jun inducing a transient upregulation of cell cycle division 2 (Cdc2) in the dedifferentiated SCs [24]. Cdc2 in turn phosphorylates vimentin that, via β1 integrin, interacts with basal lamina proteins such as laminin. The latter proteins are secreted and deposited in the extracellular matrix by the SCs themselves [25,26]. The basal membrane proteins on the elongated SCs mediate interactions between SC integrin and the growth cone adaptor molecules on the growth cone [26]. Thereby they promote axonal elongation into and along the endoneurial tubes of the distal nerve stumps, leading toward the denervated targets [2,17,26,27,28]. The process of axonal outgrowth and entry into the endoneurial tubes is, however, both complex and tardy especially after surgical repair of the transected nerve stumps (Figure 2C–I) [29,30].

### 1.3. Challenges to Functional Recovery after Nerve Injury

Despite microsurgical skills in nerve repair, the regenerating axons fail to select their original endoneurial tubes [31]. The resulting misdirection of regenerating nerve fibers to reinnervate targets that they did not supply formerly, remains one of the biggest challenges for functional recovery after peripheral nerve injuries [6,32,33].

**Figure 3:**
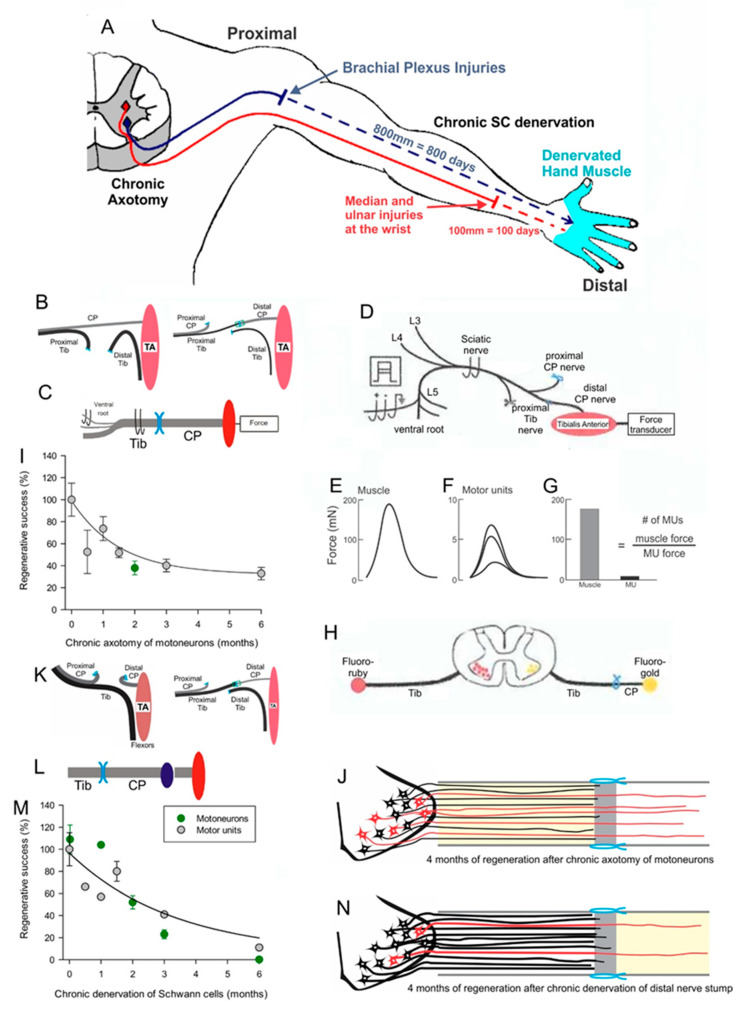
A. Motoneurons remain without targets (chronic axotomy) as they regenerate after proximal nerve injuries such as brachial plexus injuries and Schwann cells (SCs) in the denervated distal nerve stumps remain chronically denervated. At the slow rate of 1 mm/day regeneration rate in human, a year or more will pass for regenerating nerves to reach hand muscles, which are generally believed to atrophy and be replaced by fat (**A**). To isolate the effects of chronic axotomy from chronic denervation, the tibial (TIB) nerve was cut and the stumps sutured to innervated muscle prior to a cross-suture of TIB nerve to freshly denervated common peroneal (CP) distal nerve stump (**B**) and tibialis anterior (TA) muscle reinnervation was assessed by recording muscle and motor unit (MU) isometric forces at least 4 months later (**C**), as shown in more detail in (**D**). After cutting all nerves other than the CP nerve to the TA muscle, the sciatic nerve was stimulated at 2x threshold to evoke and record isometric muscle twitch (and tetanic—not shown) force (**E**). Thereafter, ventral root filaments were dissected to stimulate, at 2x threshold, single CP nerve fibers and record all-or-none MU twitch forces (**F**). The number of MUs was determined by dividing TA muscle force by average TA MU forces to give the number of TIB nerves that reinnervated the TA muscle (**G**). Numbers of axotomized motoneurons regenerating axons into the freshly denervated CP nerve stumps were also counted after their labelling by applying retrograde dyes, either fluororuby or fluorogold, to the distal nerve stump 10 mm from the cross-suture site (**H**). Plots of the numbers of reinnervated MUs and motoneurons regenerating their axons as a function of the days of axotomy prior to TIB-CP cross-suture, demonstrated an exponential decline to 33% of the numbers after immediate cross-suture at 0 days (**I**). The outgrowth of axons across the suture site is shown diagrammatically in (**J**). In order to examine the effects of chronic Schwann cell denervation, the CP nerve was transected and the proximal stump sutured to innervated muscle before the TIB-CP cross-suture of the freshly cut TIB nerve to the chronically denervated CP nerve stump to record muscle and MU forces (**K**) or to apply retrograde dye to the regenerated nerve 10 mm from the suture site (**L**). The numbers of reinnervated MUs and TIB motoneurons that regenerated their axons into the CP nerve stump, declined exponentially to <5% within 6-months of CP distal nerve chronic denervation (**M**), as illustrated figuratively in (**N**). Adapted from [35,36].

A second major challenge to functional recovery after nerve injuries is (1) the decay in the neuronal regenerative capacity after chronic axotomy, the state in which the neurons are regenerating their nerves but have not yet made functional contact with their denervated targets (Figure 3B–J) [20,34,35] and (2) the severe decline in axonal regeneration through chronically denervated nerve stumps (Figure 3K–N) [20,35,36]. The chronically denervated SCs progressively lose their growth supportive phenotype (Figure 1C,D,H–M) and their numbers decline [19,33,37,38,39]. The chronically denervated muscle fibers retain their satellite cells that contribute nuclei as the fibers recover size after reinnervation. They also contribute more nuclei when the denervated fibers are electrically stimulated and thereby, reduce denervation atrophy of the fibers [40]. Nonetheless, the failure of chronically denervated muscle fibers to recover their former size fully, suggests that there may be a limit to the number of the proliferative capacity of the satellite cells [35].

### 1.4. Promising Strategies to Improve Nerve Regeneration

The majority of human nerve injuries, resulting from vehicle accidents, recreational activities and iatrogenic injuries during surgeries, occur in the upper extremity (Figure 3A) [42]. Seminal studies in rodents in the Gordon and Brushart laboratories with Canadian and German collaborators [29,43,44,45,46,47] (reviews [33,48,49,50,51,52]), and in human patients in the Chan laboratory in Canada [53,54,55] (reviews [33,48,52,56]), demonstrated that brief low frequency, electrical stimulation of the nerve proximal to the site of transection and surgical repair, accelerated motor and sensory nerve regeneration. More recently, a positive conditioning effect of electrical stimulation of the intact nerve, prior to nerve transection and repair, has been pursued in rat studies in the laboratories of Chan and Webber [57,58,59,60].

There is also a long history of surgical repair via autologous nerve bridges and via a wide variety of conduits that include acellular allografts [61,62,63,64]. Other important surgical procedures in practice are (1) the nerve transfer of either a redundant proximal nerve stump or dissected fascicles of a functioning nerve to a denervated distal stump to restore function [65,66,67,68,69] and (2) the transfer of tendons to assist lost movements [70]. The use of stem cells such as mesenchymal stem cells as substitutes for SCs is also being explored in animal models [71,72].

### 1.5. The Scope of this Review

This review concerns specifically (1) the processes of axon outgrowth from proximal to distal nerve stumps, (2) the growth of these axons in the stumps and within the intramuscular nerve pathways to target denervated muscle fibers, (3) the selection and number of muscle fibers that are reinnervated by the regenerated motor nerve fibers and (4) the recovery of the properties of the nerve and muscle fibers after muscle reinnervation. The reinnervation fate of sensory nerve targets is not considered here.

## 2. Axon Outgrowth and Regeneration

### 2.1. Neuronal and Schwann Cell Responses to Injury

After nerve injuries that isolate the distal stump from the neuronal soma, the proximal nerve stump degenerates to the first node of Ranvier prior to sealing the membrane within hours [17,73,74,75,76]. This sealing is facilitated by calcium released from intracellular stores, activating proteases such as calpain to cleave cytoskeletal actin and spectrin and thereby, reducing membrane tension and accelerating the membrane fusion [76]. The calcium signaling, via phosphorylation of mitogen-activated protein kinase (MAPK) and Ca^2+^/calmodulin-dependent protein kinase (CaMK), stimulates retrograde signaling to activate expression of injury responsive genes in the neuronal soma and, in turn, axonal outgrowth [77,78,79]. Within axons proximal to the injury, the robust de novo synthesis of rapamycin (mTOR) is mirrored by the decline in the synthesis of the functional mTOR antagonist, PTEN [80]. The mTOR mRNA from the cell body binds to nucleolin for anterograde transport by the kinesin motor to the injury site. There it is translated locally [75]. In the axon, the mTOR protein controls its own mRNA translation and that of many localized mRNAs. These include the transcription factors that bind to dynein via adaptor proteins such as importin β1, to transport them retrogradely to the neuronal nucleus where they, in turn, regulate transcriptional responses [75,80].

After nerve injury, the gene expression in axotomized neurons and in denervated distal nerve stumps has been compared and contrasted with that during neuronal development: hundreds of growth-associated genes [81,82] that transcribe proteins such as those shown in Figure 1A, are upregulated. Injured motoneurons, but not immature motoneurons, are considered to shift their gene expression from a transmitting to a growth mode [3,83]. Genes that transcribe proteins, including the cytoskeletal proteins, actin and tubulin, and GAP-43 which support axonal growth, are upregulated. Genes that transcribe proteins associated with chemical transmission, including choline acetyltransferase and acetylcholinesterase, are downregulated concurrently (Figure 1A). Similarly, the SCs in the distal nerve stump upregulate genes that transcribe proteins that support axon growth and concurrently downregulate myelin-associated genes.

### 2.2. Outgrowth of Axons from the Injury Site

Axon sprouts emerge rapidly from the proximal stump with the local cytoskeletal proteins available within the stump [84,85]. The proteins are replenished by their slow axonal transport following their gene upregulation and translation in the neuronal cell body [85,86,87,88,89]. Even after a nerve crush injury where the endoneurial tubes remain intact, there is a delay with latent periods of days before the axon sprouts entering into and regenerating within the denervated tubes [29]. This delay is related, at least in part, to the inhibition by remaining chondroitin sulphate proteoglycan [90,91]. The inhibition is overcome as the regenerating axons grow between the extracellular matrix of the basal lamina containing growth-promoting molecules, including fibronectin and laminin, and the layer of elongated SCs that infiltrate the injury site and along the denervated nerve stump (Figure 2B) [2,92,93].

After injuries that disrupt the endoneurial tubes, including transection injuries, the migration of SCs into the injury site after surgical repair is time-consuming. The axon sprouts “wonder” within the space between proximal and distal nerve stumps, branch, and even grow back into the proximal nerve stump (Figure 2D,F,I) [41,94]. As a result, the progress of regenerating axons into the distal nerve stumps is slow or “staggered”, as demonstrated by immunostaining and silver chloride staining (Figure 2E,G,I). Application of a fluorescent dye just distal to the suture site revealed a month-long period for all the motoneurons to regenerate their axons across the suture site and into the distal nerve stump (Figure 2A–C) [29].

## 3. Axon Regeneration into Distal Nerve Stumps

### 3.1. Schwann Cell Neurotrophic Factors and Preferential Reinnervation

**Figure 4:**
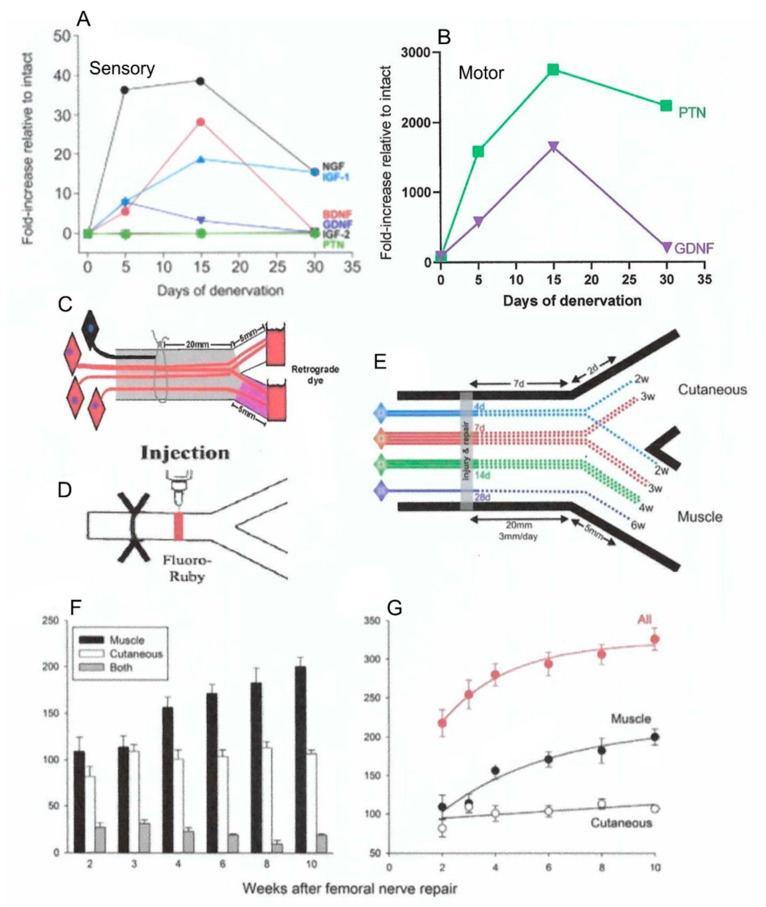
The expression of neurotrophic factors in Schwann cells in denervated nerve stumps and their role in preferential reinnervation of appropriate pathways by regenerating femoral nerves. The time course of the gene expression measured with rtPCR in the cutaneous sensory nerve branch of the femoral nerve (**A**) and in ventral root motor nerves (**B**). The mRNA normalized to values in innervated nerve stumps increased to a peak at 15 days and receded thereafter. Two different retrograde dyes (fluorogold and rubyred) were applied for retrograde labelling and counting of the motoneurons that regenerated their axons into appropriate and inappropriate motor (muscle) and sensory (cutaneous) nerve branches (**C**), and 1.5 mm across the site of microsurgical repair of the transected femoral nerve (**D**). The regeneration of the motor axons is non-selective at 2 and 3 weeks for the appropriate and inappropriate motor and sensory femoral nerve branches (**E**). However, as also shown by the data in (**F**,**G**), motoneurons progressively regenerate their axons preferentially into the motor branch in the time frame in which Schwann cells in the sensory and motor nerve branches show selective expression of growth factors (**A**). The number of motoneurons that had regenerated their axons non-selectively into both branches did not change over time (**F**,**G**). Adapted from [33,98,99].

The SCs that myelinate intact sensory nerves or encase the non-myelinated small sensory fibers, begin to express sensory-specific neurotrophic factors within 5 days of denervation; the SCs that normally myelinate motor nerve fibers, begin to express their motor specific neurotrophic factors (Figure 4A,B) [95,96,97]. The mRNA levels of these factors have not reached their peak levels in the motor SCs approximately (~) 10 days after nerve repair when ~50% of the femoral motoneurons regenerate their axons across the suture site (Figure 2C and Figure 4B) [29]. Two and three weeks later, the motoneurons regenerate their axons randomly into the appropriate motor and inappropriate sensory branches with few motoneurons regenerating their axons into both branches (Figure 4D,F) [98]. Once the mRNA levels of the motor-specific neurotrophic factors reach peak values by 15 days, *all* the remaining axotomized motoneurons regenerate their axons into the appropriate motor branch progressively over an 8 to 10 week period (Figure 4E–G). The number of the femoral motoneurons that regenerate their axons into the inappropriate sensory nerve branch, remains stable from the time point of three weeks when the reinnervation was random (Figure 4F,G). This provides further evidence for the specific selection by the motoneurons to regenerate into appropriate motor pathways containing SCs that express motor specific neurotrophic factors. Sensory neurons also demonstrated the same delayed specificity of reinnervation of the appropriate cutaneous nerve [44] in line with the concurrent SC expression of the sensory-specific neurotrophic factors (Figure 4A). Hence, the time course of expression of neurotrophic factors specific to motor and sensory SCs accounts for the initial random reinnervation of appropriate nerve branches and their subsequent preferential reinnervation of appropriate nerve branches [99].

### 3.2. Failure of Regenerating Motor Fibers to “Find” Their Former Muscle and Muscle Fibers

**Figure 5:**
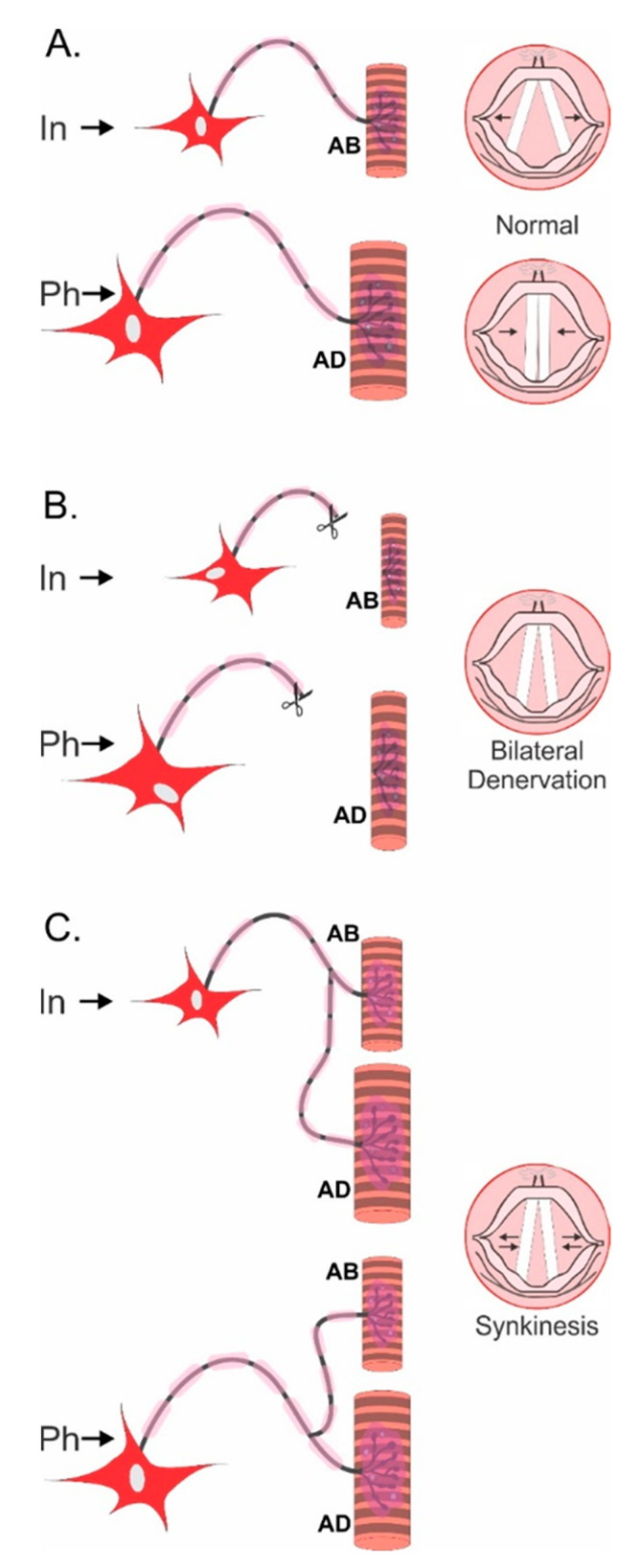
**Non-selective reinnervation of muscles after peripheral nerve surgery.** The diagrammatic representation of the innervation of the intrinsic abductor (AB) and adductor (AB) muscles of the vocal cords by inspiration and phonation (speaking) in normal (**A**), denervated (**B**), and reinnervated (**C**) intrinsic muscles. The large and small motoneurons innervating the AB and AD muscles, respectively, represents the 4:1 ratio of the number of the motoneurons innervating the AB and AD muscles. After bilateral denervation, the vocal cords do not close during phonation so that speech is severely impaired (**B**). After surgical repair of the laryngeal nerve, the axotomized motoneurons randomly reinnervate the AB and AD muscles (**C**) with the result that the vocal cords are effectively paralyzed in a position of openness that disallows effective speech. Adapted from [104].

In spite of the specificity of motor and sensory nerves for their appropriate pathways, the pattern of reinnervation of denervated muscles reveals the failure of regenerating motor nerve fibers to “find” the appropriate motor pathways that lead them back to the muscle fibers that they had innervated previously [100,101]. It is this misdirection that is a major contributor to the long-term functional deficits which are seen after surgical repair of nerve injuries [101,102,103]. An example of this misdirection is the random reinnervation of denervated hand muscles that was demonstrated in patients after surgical repair of a transected ulnar nerve at the wrist, and the resulting in co-contraction of antagonistic muscles with loss of fine movements [32]. Another example is the misdirection of regenerating laryngeal nerves that frequently leads to an apparent paralysis of the adductor and abductor muscles of the vocal cords that, in turn, results in the lost ability of the patients to speak (Figure 5) [104].

Should a common nerve supplying agonist muscles be injured and surgically repaired, the recovered movements may appear to be appropriate despite the random reinnervation of the denervated muscles by the regenerated nerve fibers. Such a case was demonstrated experimentally when the reinnervation of the lateral gastrocnemius and soleus muscles was shown to be random after transection and surgical repair of the common LGS nerve [101]. Yet, there is an interesting experimental observation of regenerating nerve fibers after surgical repair of the transected common peroneal nerve, displaying the spatial preferences shown by the normal innervation that locates slow- and fast-twitch muscle fibers to the deep and more superficial regions of the rat tibialis anterior muscle [105]. This was the case even though the obvious clumping of the muscle fiber types within the regions demonstrated that the regenerating nerve fibers did not find their original muscle fibers.

## 4. Regeneration within Intramuscular Pathways and Muscle Reinnervation

### 4.1. Motor Unit Territories in Normally Innervated Muscles

**Figure 6:**
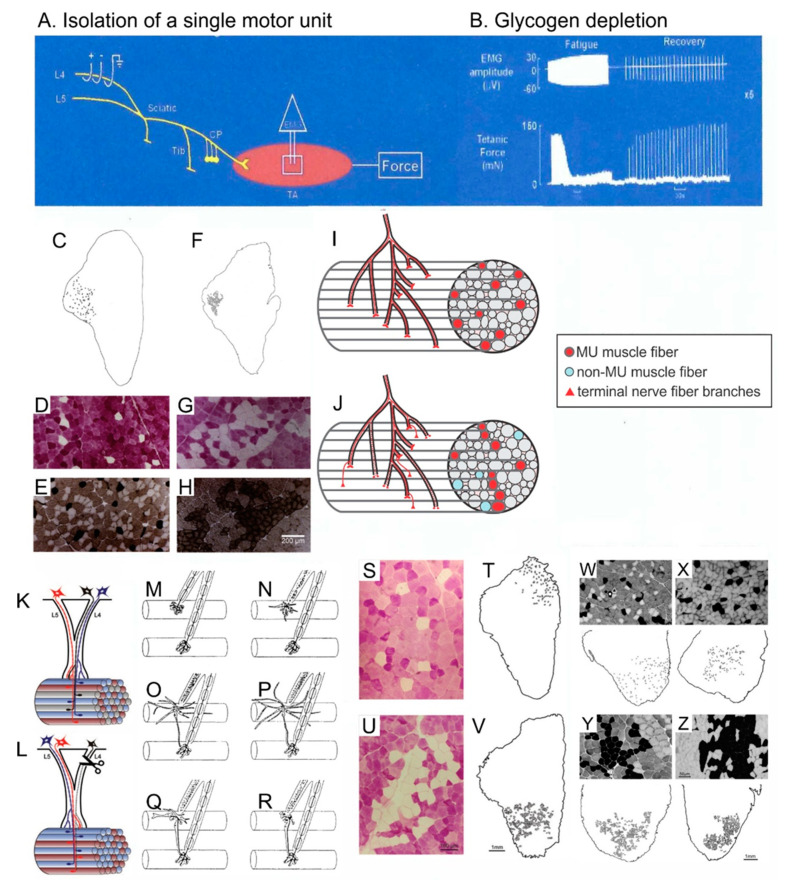
The motor unit (MU) spatial territory that contains all the muscle fibers innervated by one motoneuron, limits the numbers of muscle fibers that each reinnervating nerve can reinnervate after complete and partial nerve injuries. Muscle force was recorded from isolated MUs in tibialis anterior (TA) muscle by all-or-none electrical stimulation of dissected ventral root filaments after a laminectomy to expose the lumbosacral spinal cord (**A**). The MU muscle fibers were depleted of their glycogen by repetitive stimulation at 1 and 5 Hz to fatigue the MU fibers followed by recovery during 0.1 Hz stimulation This sequence was repeated until the MU force failed to recover (**B**). The TA muscle was dissected rapidly. The muscle was frozen for cryosectioning and staining for glycogen with Periodic Schiff reaction to reveal and count glycogen depleted MU fibers in normally innervated (**C**,**D**) and reinnervated (**F**,**G**) TA muscle fibers after common peroneal (CP) nerve section and surgical repair and for histochemical staining of acidic mATPase to fiber type the MU muscle fibers (**E**,**H**). The MU muscle fibers are normally distributed in a mosaic pattern (**C**–**E**) which changes to F, G, H. clumping of the fibers that occupy a smaller territory (**F**–**H**). The branching pattern of the motor nerve is shown in normally innervated (**I**) and reinnervated (**J**) MUs. In normally innervated muscles, each motor nerve normally branches only once the nerve enters the muscle. Thereafter, the nerve branches to distribute the fibers to several fascicles to give the mosaic pattern of MU muscle fibers (**I**). In reinnervated muscles, the loss of the mosaic pattern reveals that the regenerating nerves “miss” some of the denervated intramuscular nerve sheaths and branch more as they approach the muscle fibers, often within single fascicles. This gives rise to the clumping of reinnervated muscle fibers (**J**). On the other hand, the normal mosaic distribution (**I**,**K**) is progressively lost after partial muscle denervation by transecting one of two contributing ventral roots (**L**). Diagrammatically, the peri-synaptic Schwann cells at the endplates (**M**,**N**) begin, after partial denervation of the muscle, to extend processes (**O**) some of which extend to the innervated endplate of an adjacent muscle fiber (**O**,**P**). The processes lead nerve sprouts from innervated to denervated endplates to reinnervate the denervated muscle fibers (**Q**,**R**). Normally innervated (**S**,**T**,**W**) and partially denervated (**U**,**V**–**Z**) MU muscle fibers are compared to show the progressive MU fiber and muscle fiber type-grouping that occurs as the percentage of remaining MUs declines from 100% (normal; **W**), to 60% (**X**), 30% (**Y**), and 15% (**Z**) in partially denervated muscles. Adapted from [5,109,121].

Motor nerves do not branch during their course from their cell bodies in the ventral spinal cord to their destination of a skeletal muscle until they enter and branch within the intramuscular nerve sheaths of that muscle [106,107]. The branching resembles the branching of a tree above and below the ground, the nerve and the sheath branching progressively and thereby, guiding the nerve branches to different muscle fascicles. As a result, the muscle fibers innervated by one motoneuron in a motor unit (MU), occupy a discrete “territory” in the muscle cross-section (Figure 6C,F) [105,108,109,110]. This branching pattern was visualized in individual nerves in transgenic mice [107]. Earlier in 1992, it was demonstrated in normally innervated and reinnervated muscles after partial and complete nerve injuries, by depleting the muscle fibers of single MUs of glycogen (Figure 6A–C) [105,106,110,111]. The procedure of glycogen depletion is to isolate and perform repetitive bouts of tetanic electrical stimulation of a single MU to fatigue its muscle fibers (Figure 6A,B). Thereby, the MU muscle fibers are depleted of glycogen and visualized as white with the periodic Schiff reaction (Figure 6D,G,S,U). The spatial analysis of the MU muscle fibers demonstrates the outer limits of the nerve branching, the MU territory. The size of the MU territory increases in proportion to the numbers of muscle fibers in the MU, the territory occupying as much as 1/3rd of the muscle cross-sectional area [105].

### 4.2. Motor Unit Territories after Muscle Reinnervation

**Figure 7:**
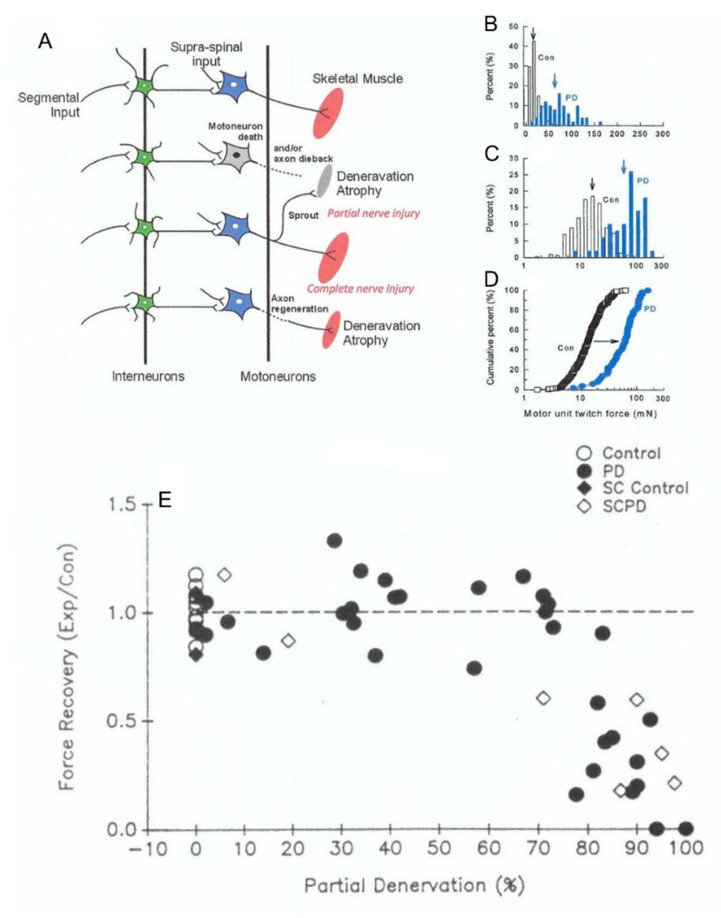
**Motor unit enlargement after partial denervation of muscles.** Figurative illustration of muscle denervation, reinnervation after complete nerve injury, and compensatory sprouting after partial nerve injury (**A**). The skewed distribution of motor unit (MU) twitch forces in control tibialis anterior (TA) muscle is shifted significantly (*p* < 0.05) to the right to larger values after partial denervation by cutting the L5 spinal root (**B**)**.** The shift is more obvious when the MU forces are plotted on a logarithmic scale (**C**) and as cumulative distributions (**D**). The cumulative distributions demonstrate that all MUs in the partially denervated muscle increase by the same factor such that the large MUs include many more muscle fibers by sprouting than the small MUs. This five-fold increase is maximum as shown in (**E**). by the sharp decline in muscle force recovery when partial denervation of the muscle (numbers of nerves innervating the muscle) exceeds about 80%. The sprouting in partially denervated muscles (PD) is the same whether the spinal cord is intact or hemisected (SCPD). Adapted from [109]_._

The normal size of the MU territories is retained after partial nerve injuries but the mosaic distribution of the muscle fibers disappears gradually with “clumping” of the MU fibers increasing inversely with the number of intact MUs (Figure 6L,W–Z) [109]. After complete nerve injury and surgical repair, the normal mosaic distribution is replaced by a “clumped” distribution of the reinnervated muscle fibers (Figure 6C–K) [105]. The “clumping” becomes progressively more extensive when the number of regenerating nerve fibers was reduced experimentally by cutting one ventral root [110]. Because each MU muscle fiber is surrounded by ~5–8 non-MU fibers, it follows that a 5–8 fold increase in muscle fibers per motoneuron should be the upper limit to which each motoneuron can reinnervate denervated muscle fibers [105]. Indeed, the increase in MU size, namely the number and contractile force of the muscle fibers supplied by each motor nerve, increases to a limit of 5–8 fold that compensates for up to ~80% reductions in MU numbers in reinnervated muscles after partial and complete nerve injuries (Figure 7) [110,112,113,114]. It is only when less than 20% of the motoneurons reinnervate the muscles that denervated muscle fibers remain and the muscle force declines (Figure 7E) [109,115].

### 4.3. Perisynaptic Schwann Cells

Non-myelinating perisynaptic SCs (PSCs) are present at the endplate region of the neuromuscular junction where the motor nerve innervates the muscle fibers [116]. The cells respond to the acetylcholine released from the nerve terminals [117] and extend processes only when neuromuscular transmission is interrupted by partial or complete muscle denervation (Figure 6K–P) [4,116,117,118,119,120,121,122,123]. After partial nerve injury, the processes bridge between innervated and denervated junctions and thereby, they guide the axon sprouts emerging either from the last node of Ranvier of the intact nerve or from the terminal itself, to reinnervate the denervated endplates of neighboring muscle fibers (Figure 6N–R) [5,121,122,124]. The progressive clumping of MU muscle fibers in partially denervated muscles is accounted for by this localized guidance of axon sprouts to neighboring denervated muscle fibers. On the other hand, the progressive clumping that occurs when the number of regenerating nerve fibers is reduced after complete injuries, may be accounted for by each regenerating nerve fiber missing “branch points” within the denervated intramuscular nerve sheathes (Figure 6J). As a result, the nerve fiber branches in the more distal regions of the sheathes, closer to the denervated muscle fibers. Finally, they are guided by PSCs to the denervated endplates [122].

### 4.4. In Summary

These findings demonstrate the many levels of misdirection of regenerating motor fibers with evidence of (1) regenerating fibers randomly entering vacant endoneurial tubes at the site of nerve repair after transection injuries and the consequent misdirection of the fibers to different denervated muscles, even to physiologically antagonist muscles, (2) motor and sensory neurons regenerating their axons into inappropriate channels initially prior to channeling their axons appropriately once neurotrophic factors are differentially expressed by SCs in the sensory and motor endoneurial tubes, and (3) regenerating motor fibers branching within the intramuscular sheaths but “missing” branch points of the sheaths and reinnervating muscle fibers that they did not supply previously.

## 5. Recovery of the Reinnervated Nerve and Muscle Properties

### 5.1. Nerve–Muscle Size Relationships

**Figure 8:**
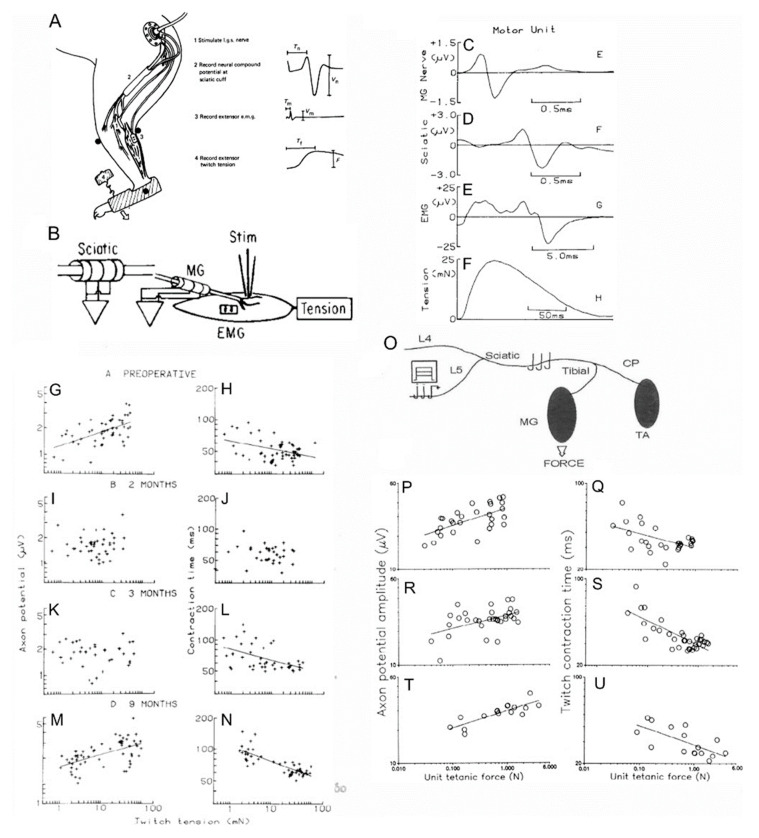
**Size relationships in cat medial gastrocnemius (MG) muscles after complete and partial nerve injuries.** At regular intervals of 10–14 days, the cat was anesthetized with fluorothane. The foot of the operated hindlimb was encased in a special boot that was attached to a force transducer to record isometric muscle and motor unit (MU) forces in response to stimulation of the MG nerve via nerve cuff electrodes (**A**) and in response to stimulation of single MG nerves via a needle electrode inserted through the skin into the endplate region of the MG muscle (**B**). The MU action potentials on the MG (**C**) and sciatic (**D**) nerves were recorded from implanted nerve cuff electrodes. The MU electromyographic action potential (EMG; **E**) was recorded from EMG pad electrodes on the muscle surface and the twitch tension (**F**) was recorded with the force transducer attached to the boot. MU action potential amplitude was directly correlated with the twitch tension of the innervated muscle fibers (**G**) and inversely correlated with the contraction times of the twitch contractions (**H**). These correlations are in accordance with Henneman’s size principle (see text). The inverse relationship demonstrates that force increases as contraction times become shorter with the fast MUs being the most forceful and the slow MUs being the least. Early during reinnervation, the size relationships were lost when each nerve reinnervated muscles fibers of different histochemical types(**I**,**J**) but they returned with time (**K**–**N**) as the heterogenous muscle fibers were respecified by their new motoneuron innervation (not shown). In normal (**O**,**P**) and partially denervated (**R**–**U**) MG muscles after transecting the L7 root, the size relationships were retained as the forces increased when the partial denervation removed 50% and 80% of the nerve supply to the muscles. All correlations in which lines are drawn were statistically significant at the 0.05 level. Adapted from [112,125,133].

The time course of recovery of nerve–muscle properties after muscle reinnervation was determined in experiments in which chronic recordings were made in each animal before and over time, after nerve transection and surgical repair. Muscle and MU forces were elicited under fluorothane anesthesia, by stimulating the motor nerve and single nerve fibers intramuscularly with a bipolar needle electrode, respectively (Figure 8A,B,F) [125]. The forces were recorded at regular intervals prior to and following MG nerve transection and surgical repair, by placing the cat foot in a boot without compromising blood flow and coupling the boot to an isometric force transducer. The muscle and MU electromyographic signals (EMG) were recorded with sheet electrodes on the muscle surface (Figure 8A,E). Compound and MU nerve action potentials recorded on cuff electrodes on the sciatic nerve and on the MG and sciatic nerves, respectively (Figure 8A,C,D).

A positive correlation between MU twitch forces and MU nerve action potential amplitudes (Figure 8G), and a negative or inverse correlation between MU twitch forces and their contraction times (Figure 8H), were demonstrated, confirming the size relationships that are the basis for Henneman’s size principle of orderly recruitment of MUs from small to large during movement [126,127,128]. These size relationships are lost initially after nerve transection and repair (Figure 8I,J) but they return with muscle force increasing with nerve size as measured by the nerve MU action potential amplitude, and inversely correlated with the MU contraction time (Figure 8L–N) [115,125,129,130]. Moreover, the reinnervated MUs are recruited in order of their size during movement as they are normally in animals [131] and humans [32]. Return of the size relationships was also demonstrated in muscles reinnervated after partial denervation in experiments in which nerve filaments were dissected to record MU properties in final experiments under deep Nembutal anesthesia (Figure 8O–U) [112]. The MU force increases with the number and cross-sectional areas of the component muscle fibers in both normal and reinnervated muscles in rat [108] and cat [132] hindlimbs. Hence, the return of the size relationship between force and nerve size in reinnervated muscles (Figure 8G,M,P,T) and their orderly recruitment of MUs during movement, demonstrates that Henneman’s size principle applies to both normal and reinnervated muscles, namely, nerve size controls the number of muscle fibers innervated by each motoneuron and MUs are recruited in order of their size [126,127,128].

### 5.2. Reversal of Nerve and Muscle Fiber Atrophy after Reinnervation

The size relationships are lost during the initial stages of reinnervation (Figure 8I,J) as the size of the nerves proximal to the site of transection and surgical repair declines after axotomy [125,133,134,135,136,137]. In accordance with the nerve fiber size being determined by neurofilament content [138,139], the decline in size is accompanied by the downregulation of neurofilaments in the axotomized motoneurons (Figure 1A). The other cytoskeletal proteins, tubulin and actin, are upregulated and transported to the outgrowing axon sprouts that regenerate into and within the distal nerve stumps (Figure 1A,E) [2,3,20,33].

Denervated muscle fibers atrophy prior to their reinnervation such that they generate less force at the early stages of reinnervation (Figure 8I,J) [125,129]. The MU muscle fibers are normally homogeneous with respect to their metabolic properties and myosin content [140,141,142]. In contrast, reinnervated MU muscle fibers after complete and partial nerve injuries are heterogeneous because the regenerating nerves reinnervate muscle fibers that they did not formally innervate (Figure 6C–J,S–Z) [105,108]. As the size relationships return in the reinnervated muscles, the nerve fibers recover their normal size [125,143,144]. The range in size of MU muscle fibers of each type overlap rather than their normal ranges increasing from the slow to the fast fiber types [145]. It is the size-dependent branching of the regenerating nerves in the denervated intramuscular nerve sheaths that restores the MU forces. Thereby, this branching re-establishes the direct correlations of the size of the nerves and their MU forces.

Delayed nerve repair and the nerve injuries that are suffered far from their target contact (Figure 3A), impact the full recovery of the size of denervated muscle fibers [35,36,146]. On the other hand, they do not prevent the full recovery of the caliber of the nerve fibers that do succeed in remaking functional contacts with denervated targets [144].

### 5.3. Activity-Related Specification of Muscle and Motoneuron Properties

It is likely that the pattern of neuronal activation plays an important role in the re-specification of muscle properties after nerve regeneration and muscle reinnervation. This is because electrical stimulation of all motor nerves supplying normally innervated muscles transforms the properties of the muscle fibers [141,142,147]. Stimulation for less than 5% of each day converts the normally heterogenous muscle fibers to fast glycolytic fibers, stimulation for 5% of each day to fast oxidative glycolytic fibers, and 50% per day stimulation to slow oxidative fibers [148,149,150]. These histochemically identified muscle fibers correspond with physiologically identified MUs, classified according to their contractile speed, presence or absence of the sag of unfused tetanic contractions, and fatigability into fast fatigable, fast fatigue-resistant, and slow fatigue-resistant MUs [111,151,152]. Consistent with the stimulation at 50% of each day transforming muscle fiber properties to the slow type, the 50% daily stimulation also transformed all MUs to a homogenous population of slow MUs [153]. Importantly, the electrical stimulation also transformed the properties of the motoneurons with conversion to the corresponding motoneuron types [154]. Hence, the re-specification of nerve and muscle properties after nerve injuries likely involves the re-establishment of the normal recruitment of the MUs from small slow MUs to the largest fast MUs. Indeed, the normal order of recruitment has been documented in human reinnervated muscles after complete and partial nerve injuries [32,155]. However, the misdirection of regenerating nerves to reinnervate several different muscles interferes with the normal use of the reinnervated muscles, especially in the hand where fine movements may be lost after nerve injuries that severe the nerves to the hand muscles [32].

### 5.4. In Summary

The neuromuscular plasticity displayed after nerve injuries and reinnervation of muscles is impressive, observing many of the same principles of nerve–muscle interaction. However, it may be insufficient to restore fine movements because of the considerable misdirection of regenerating nerve fibers after transection injuries. In addition, the considerable delay in muscle reinnervation severely compromises the regenerative capacity of the axotomized motoneurons and the growth support provided by denervated SCs in the distal nerve stumps.

## 6. Conclusions

Obstacles to the recovery of function after peripheral nerve injuries remain. This is despite the considerable and impressive plasticity of the neuromuscular system that observes many of the normal principles of nerve–muscle interactions during nerve regeneration and muscle reinnervation. The primary obstacles include (1) the misdirection of regenerating motor nerve fibers to muscles and their muscle fibers which they did not formerly supply. This misdirection results from the random reinnervation of the denervated endoneurial tubes in the distal nerve stump and intramuscular nerve sheaths, respectively, and (2) the progressive regression of the expression of growth-associated genes in the axotomized neurons and in the supporting denervated SCs. This regression accompanies a progressive failure of regenerative success after delayed nerve surgeries and/or proximal nerve injuries that require nerves to regenerate over long distances to reinnervate distant denervated target organs. These obstacles continue to confront the clinicians who repair peripheral nerve injuries. Nonetheless, there are several surgical manipulations in clinical use that do promote functional recovery. These include nerve and tendon transfers, and the potential for brief low-frequency electrical stimulation of the proximal nerve stump at the time of nerve repair to accelerate the outgrowth of axons and their crossing of the suture site of proximal and distal nerve stumps, even when the stumps require artificial and nerve grafts to connect the stumps. Hence, research and practical application of new techniques are improving the outcomes of surgical repair of injured nerves. The research is also elucidating the underlying molecular basis of nerve regeneration and the bases for the improved nerve regeneration after electrical stimulation and FK506 local application.
